# Refractory Gastroparesis in Ehlers-Danlos Syndrome: Failure of Pyloric-Directed Therapy Despite Normal Pyloric Physiology

**DOI:** 10.7759/cureus.116640

**Published:** 2026-09-21

**Authors:** Katherine Stevens, Michael Herman

**Affiliations:** 1 Osteopathic Medicine, Lincoln Memorial University, Knoxville, USA; 2 Gastroenterology, Borland Groover, Jacksonville, USA

**Keywords:** autonomic dysfunction, botulinum toxin, dysmotility, ehlers-danlos syndrome, endoflip, enteral nutrition, gastric electrical stimulation, gastroparesis, pyloric dysfunction

## Abstract

Gastroparesis is increasingly recognized in patients with Ehlers-Danlos syndrome (EDS), often driven by underlying autonomic dysfunction rather than pyloric pathology. We present a case of severe, refractory gastroparesis in a patient with EDS requiring post-pyloric enteral feeding and demonstrating no response to pyloric-directed therapy despite normal pyloric physiology on EndoFLIP. The patient ultimately improved following placement of a gastric electrical stimulator and optimization of enteral feeding strategies. This case highlights the importance of physiologic assessment in gastroparesis and demonstrates an association between normal pyloric physiology and limited response to pyloric-directed therapy in the setting of underlying neuropathic dysmotility.

## Introduction

Ehlers-Danlos syndrome (EDS) is a connective tissue disorder associated with a wide spectrum of gastrointestinal manifestations, including disordered motility [1]. When patients with EDS develop gastroparesis, the underlying mechanism is not always apparent: delayed gastric emptying can reflect a mechanical problem localized to the pylorus, a diffuse process driven by autonomic and enteric neuropathy, or both [1,2]. This distinction matters because it determines which interventions are likely to be effective. Pyloric-targeted therapies, such as intrapyloric botulinum toxin injection, have been used in some patients with refractory gastroparesis, although evidence supporting their routine use remains limited and inconsistent [3,4]. We present a case that illustrates why identifying the underlying mechanism of delayed gastric emptying, rather than treating the pylorus by default, is critical to selecting effective therapy [5,6].

## Case presentation

A 28-year-old woman with a history of Ehlers-Danlos syndrome (EDS) and mast cell activation syndrome was referred for evaluation of progressive upper gastrointestinal symptoms, including early satiety, nausea, vomiting, and poor oral intake. Her symptoms resulted in clinically significant weight loss, ultimately requiring enteral nutritional support with a nasoenteric feeding tube (DHT) placed for post-pyloric feeding. She was maintained on nocturnal tube feeds at 120 mL/hr with limited oral intake.

At presentation, she was afebrile and hemodynamically stable. Routine laboratory testing showed mild hypokalemia, likely related to poor oral intake and vomiting, without anemia, leukocytosis, renal dysfunction, or other clinically significant abnormalities (Table 1).

**Table 1:** Vital signs and laboratory findings at presentation ALT: alanine aminotransferase; AP: alkaline phosphatase; AST: aspartate aminotransferase; BUN: blood urea nitrogen; Ca: calcium; Cl: chloride; K: potassium; Na: sodium; TB: total bilirubin; WBC: white blood cell count.

Parameter	Value	Reference
Temperature	36.8 °C	36.1–37.2 °C
Blood pressure	108/68 mmHg	90–120/60–80 mmHg
Heart rate	88 beats/min	60–100 beats/min
Respiratory rate	16 breaths/min	12–20 breaths/min
Oxygen saturation	99% on room air	≥95%
WBC	6.8 ×10³/µL	4.0–11.0 ×10³/µL
Hemoglobin	12.6 g/dL	12.0–16.0 g/dL
Hematocrit	38.1%	36.0–46.0%
Platelets	264 ×10³/µL	150–400 ×10³/µL
Na	138 mmol/L	135–145 mmol/L
K	3.3 mmol/L	3.5–5.1 mmol/L
Cl	102 mmol/L	98–107 mmol/L
Bicarbonate	25 mmol/L	22–29 mmol/L
BUN	11 mg/dL	7–20 mg/dL
Creatinine	0.72 mg/dL	0.50–1.10 mg/dL
Glucose	91 mg/dL	70–99 mg/dL
Ca	9.2 mg/dL	8.6–10.2 mg/dL
Albumin	4.0 g/dL	3.5–5.0 g/dL
AST	19 U/L	10–35 U/L
ALT	17 U/L	7–35 U/L
AP	72 U/L	35–104 U/L
TB	0.5 mg/dL	0.2–1.2 mg/dL

Initial diagnostic evaluation included a gastric emptying study (GES), which confirmed delayed gastric emptying consistent with gastroparesis [5,7]. She was managed with standard medical therapy; however, symptoms remained refractory, with persistent intolerance to oral intake and ongoing nutritional challenges. She also had associated constipation, with bowel movements every 3-4 days, managed with linaclotide and prucalopride.

Given refractory symptoms, further physiologic assessment was pursued using the endolumenal functional lumen imaging probe (Medtronic Endoflip™ 300 impedance planimetry system with FLIP™ Topography [EF-300]; EF-325N 8-cm catheter; Medtronic, Shoreview, MN, USA). EndoFLIP evaluation demonstrated normal pyloric distensibility and diameter, with a pyloric diameter of 15.4 mm and a distensibility index of 5.2 mm²/mmHg at a balloon volume of 50 mL (Figure 1) [6,8]. Despite these findings, and given the severity of her symptoms, an empiric trial of intrapyloric botulinum toxin injection was pursued via esophagogastroduodenoscopy (EGD), in keeping with common practice patterns favoring pyloric-directed therapy in refractory gastroparesis [3].

**Figure 1:**
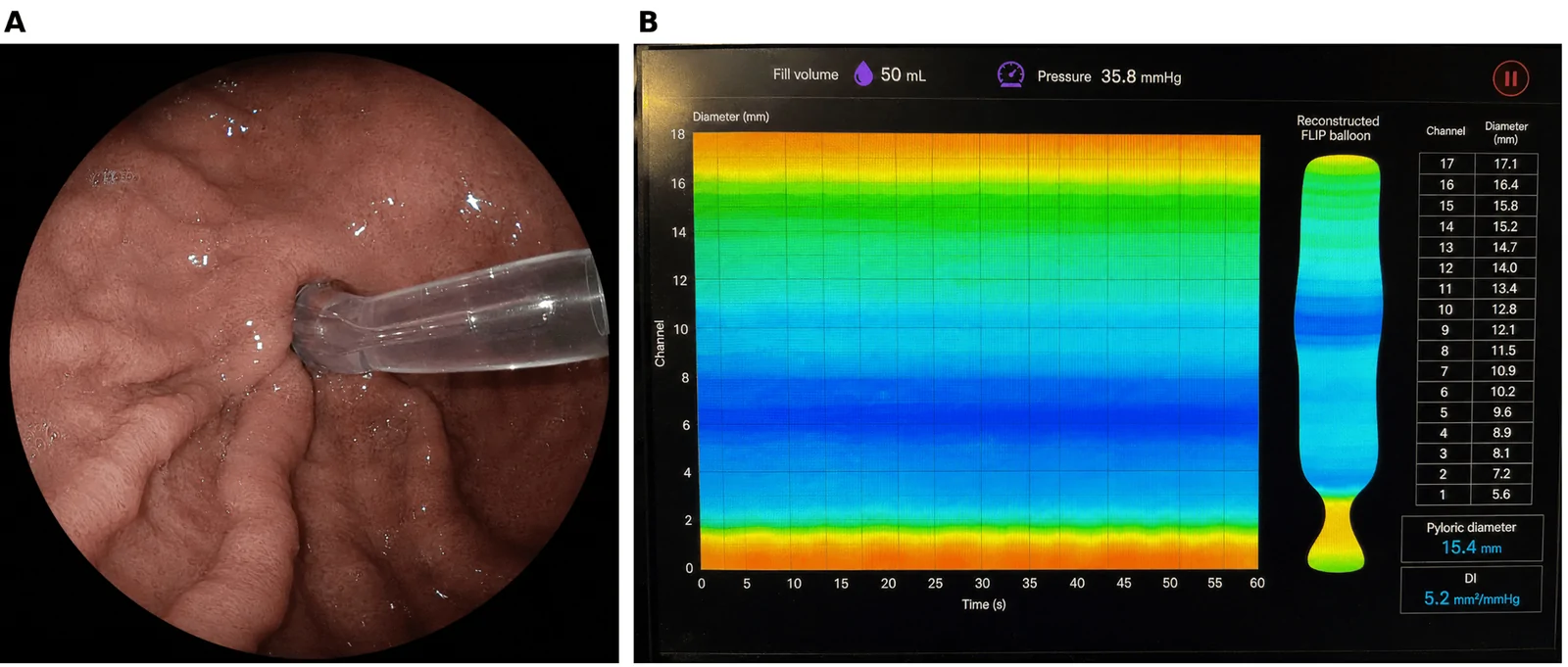
Representative EndoFLIP assessment. (A) Endoscopic illustration of the EndoFLIP balloon traversing the pyloric channel. (B) Representative output demonstrating a pyloric diameter of 15.4 mm and distensibility index of 5.2 mm²/mmHg at a balloon volume of 50 mL and intra-balloon pressure of 35.8 mmHg. EndoFLIP, endoluminal functional lumen imaging probe.

Following this intervention, she experienced no meaningful clinical improvement, with continued intolerance to both oral intake and enteral feeds, including water and electrolyte solutions.

Given the severity and refractory nature of her symptoms, she was referred for evaluation at a tertiary motility center, where further evaluation was consistent with gastroparesis and concern for more diffuse gastrointestinal dysmotility. She subsequently underwent placement of a gastric electrical stimulator. Following gastric pacemaker placement, the patient reported clinical improvement, with better tolerance of enteral feeds and oral intake and reduction in symptom severity [9,10].

During follow-up, she also reported episodic lower abdominal cramping lasting 30-45 minutes, not temporally associated with bowel movements. Adjustment of her enteral feeding regimen to a slower continuous infusion rate improved tolerance and reduced symptoms. The overall diagnostic and therapeutic course is summarized in Figure 2.

**Figure 2:**
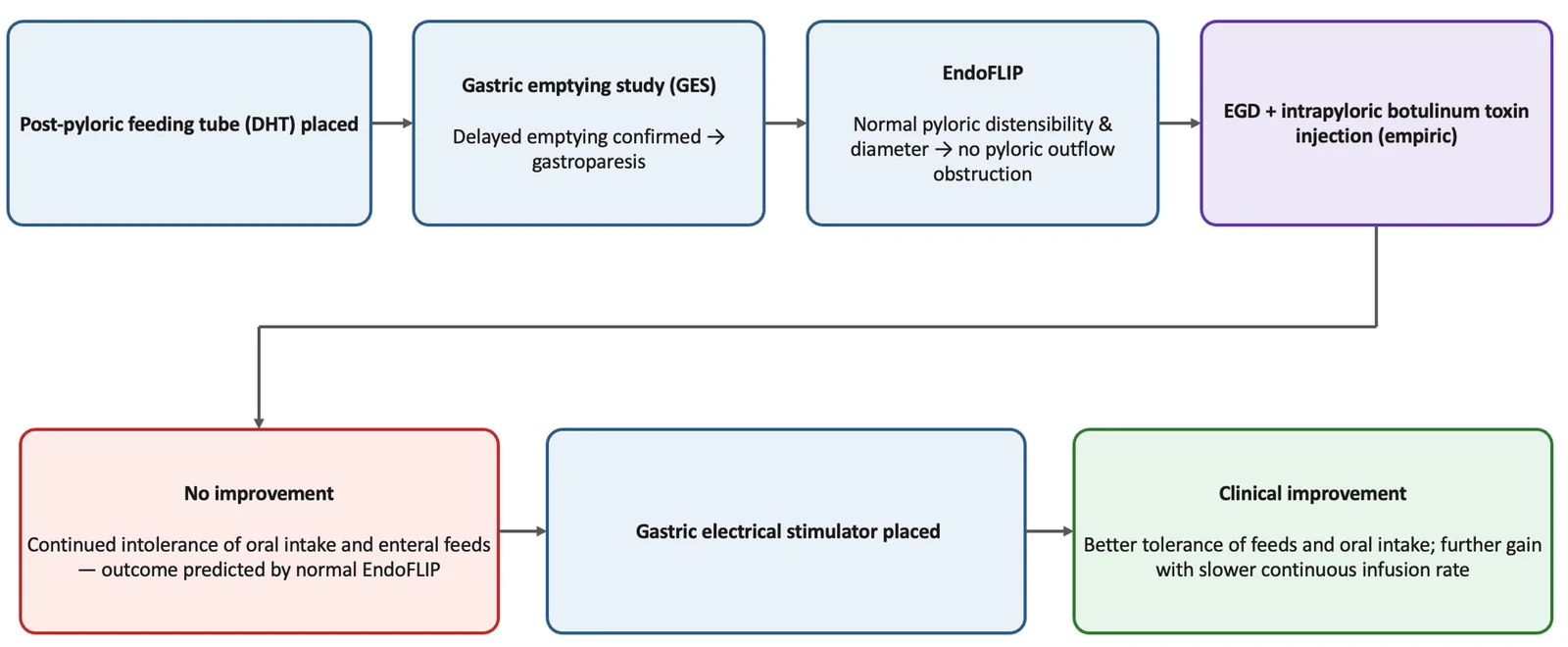
Diagnostic and therapeutic course, from initial post-pyloric feeding tube placement through gastric electrical stimulator placement and feeding-rate optimization.

## Discussion

This case highlights several important considerations in the evaluation and management of gastroparesis in patients with Ehlers-Danlos syndrome (EDS), particularly the need to distinguish between pyloric dysfunction and diffuse autonomic dysmotility.

A gastric emptying study establishes the presence and severity of delayed gastric emptying [5,7]. EndoFLIP can provide additional information regarding pyloric geometry, distensibility, and pressure-related properties. In this patient, normal pyloric distensibility and diameter did not support significant pyloric dysfunction as a predominant contributor to her symptoms [6,8].

Botulinum toxin acts by inhibiting acetylcholine release at the neuromuscular junction, reducing pyloric tone and improving gastric emptying in patients with pylorospasm or impaired pyloric relaxation [3]. In a patient with normal pyloric physiology, however, there is no physiologic target for this mechanism: the injection had nothing to correct. In this patient, normal pyloric physiology on EndoFLIP provided limited evidence of significant pyloric dysfunction, and she subsequently experienced no meaningful improvement following botulinum toxin injection.

Gastroparesis in EDS is increasingly recognized as a manifestation of autonomic dysfunction and enteric neuropathy rather than a localized pyloric problem [1,2]. Dysregulated autonomic signaling can impair fundic accommodation and antral contractility, producing delayed gastric emptying independent of pyloric tone. Her intolerance to both oral intake and post-pyloric feeds, together with her lower gastrointestinal symptoms, raised concern for more diffuse gastrointestinal dysmotility rather than an isolated pyloric abnormality.

Her improvement after gastric electrical stimulator placement supports this. Gastric electrical stimulation is thought to act on afferent signaling and central processing of nausea and gastric dysrhythmia, rather than mechanically accelerating gastric emptying [9,10].

Her feeding tolerance followed the same pattern. She initially struggled even with post-pyloric feeding at higher infusion rates, and improved once the infusion was slowed. Her feeding tolerance also improved after adjustment to a slower continuous infusion rate. This observation further demonstrates the importance of individualizing enteral feeding strategies in patients with severe gastrointestinal dysmotility.

## Conclusions

This case highlights the potential value of assessing pyloric physiology when considering pyloric-directed therapy in patients with EDS and refractory gastroparesis. In this patient, normal EndoFLIP findings were associated with limited response to pyloric-directed interventions, while clinical improvement followed gastric electrical stimulation and optimization of enteral feeding. Although a single case cannot establish a causal relationship or define an underlying mechanism, these findings illustrate the importance of considering physiologic findings alongside the broader clinical presentation when individualizing treatment for refractory gastroparesis.
